# Distinct MicroRNA Profiles in the Perilymph and Serum of Patients With Menière's Disease

**DOI:** 10.3389/fneur.2021.646928

**Published:** 2021-06-16

**Authors:** Matthew Shew, Helena Wichova, Madeleine St. Peter, Athanasia Warnecke, Hinrich Staecker

**Affiliations:** ^1^Department of Otolaryngology Head and Neck Surgery, Washington University School of Medicine in St. Louis, St. Louis, MO, United States; ^2^Department of Otolaryngology Head and Neck Surgery, University of Kansas School of Medicine, Kansas City, KS, United States; ^3^Department of Otolaryngology Head and Neck Surgery, Hannover Medical School, Hanover, Germany

**Keywords:** Meniere disease, microRNA, liquid biopsy, perilymph, biomarker

## Abstract

**Hypothesis:** Menière's disease microRNA (miRNA) profiles are unique and are reflected in the perilymph and serum of patients.

**Background:** Development of effective biomarkers for Menière's disease are needed. miRNAs are small RNA sequences that downregulate mRNA translation and play a significant role in a variety of disease states, ultimately making them a promising biomarker. miRNAs can be readily isolated from human inner ear perilymph and serum, and may exhibit disease-specific profiles.

**Methods:** Perilymph sampling was performed in 10 patients undergoing surgery; 5 patients with Meniere's disease and 5 patients with otosclerosis serving as controls. miRNAs were isolated from the serum of 5 patients with bilateral Menière's disease and compared to 5 healthy age-matched controls. For evaluation of miRNAs an Agilent miRNA gene chip was used. Analysis of miRNA expression was carried out using Qlucore and Ingenuitey Pathway Analysis software. Promising miRNAs biomarkers were validated using qPCR.

**Results:** In the perilymph of patients with Menière's disease, we identified 16 differentially expressed miRNAs that are predicted to regulate over 220 different cochlear genes. Six miRNAs are postulated to regulate aquaporin expression and twelve miRNAs are postulated to regulate a variety of inflammatory and autoimmune pathways. When comparing perilymph with serum samples, miRNA-1299 and−1270 were differentially expressed in both the perilymph and serum of Ménière's patients compared to controls. Further analysis using qPCR confirmed miRNA-1299 is downregulated over 3-fold in Meniere's disease serum samples compared to controls.

**Conclusions:** Patients with Ménière's disease exhibit distinct miRNA expression profiles within both the perilymph and serum. The altered perilymph miRNAs identified can be linked to postulated Ménière's disease pathways and may serve as biomarkers. miRNA-1299 was validated to be downregulated in both the serum and perilymph of Menière's patients.

## Introduction

Menière's disease is a chronic debilitating disorder of the inner ear, characterized by fluctuating episodes of vertigo, hearing loss, tinnitus, and aural pressure (1). Menière's disease affects 250–500 per 100,000 people and has repeatedly been shown to negatively impact patient's quality of life comparable to many other more common chronic medical diseases (2, 3). Since Prosper Menière first recognized this debilitating disease over 150 years ago, we have made significant strides to further understand the pathophysiology of Menière's disease. Most notably, 75 years ago post-mortem temporal bone analysis demonstrated endolymphatic hydrops as a pathologic correlate to Menière's disease (4). However, despite our best efforts, there remains a significant knowledge gap in the pathophysiology, diagnosis, and management of Menière's disease. While post-mortem studies have offered invaluable information and insights (5), we still have a limited understanding of what is occurring on a molecular level in patients with active Menière's disease, particularly in the early stages of the disease and other inner ear disorders with fluctuating symptoms.

The inner ear is a complex, delicate, fluid-filled structure that lies deep within the temporal bone of the skull. Unfortunately, a biopsy equivalent of the inner ear is not feasible, and diagnosis of the different diseases often rely on various forms of hearing performance testing. However, hearing performance testing such as the audiogram, do not always accurately reflect the true underlying pathology (6). As a result of these shortcomings, there is significant interest in obtaining a “liquid biopsy” equivalent of the inner ear. Many investigators are analyzing the components of inner ear fluid to gain insights into what may be occurring on a molecular level in active inner ear disease states (7–12).

Diagnosis of Menière's disease is categorized as “probable” or “definite” based on a composition of fluctuating and variable clinical criteria established by the Bárány Society and adopted by the American Academy of Otolaryngology Head and Neck Surgery (13, 14). New diagnostic methodology has aided to refine the diagnosis of Menière's disease using objective electrophysiologic testing and/or imaging, however testing is not always reliable and thus not routinely recommended (15). Unfortunately, there is no single diagnostic test for Menière's disease that serves as the gold standard. This is a result of our limited knowledge of the underlying pathology resulting in Menière's disease. Multiple pathophysiology mechanisms have been postulated including genetic (16), vascular (17, 18), immunologic (18–20), aberrant aquaporin expression (21), or a combination of causes.

Evaluation of micro RNA (miRNA) perilymph profiles in inner ear disease may help define the hearing loss on a molecular level (11). Inner ear diseases from otosclerosis to profound sensorineural hearing loss (SNHL) exhibit distinct miRNA profiles (22, 23). miRNAs are 19–23 base pair single stranded RNA sequences that regulate post translation gene expression through messenger RNA (mRNA) silencing (24). miRNA molecules are conserved throughout multiple species, have been identified in tissue and fluids throughout the body, and are known to play a significant role in various pathologies including cancer and other neurodegenerative diseases (25, 26). miRNAs have also been shown to play a key role in inner ear development and show distinct expression patterns in patients with hearing loss compared to healthy controls, making them an intriguing biomarker (27–30). In previous work, we preliminary established Meniere's disease could be diagnosed based on perilymph miRNA profile alone, further validating miRNAs as a potential biomarker either through the perilymph or serum of patients (31).

In the current study we analyzed the miRNA profiles of patients with Menière's disease, collected at the time of labyrinthectomy, and compare their profiles to patients with otosclerosis. With the goal of identifying potential Menière's specific biomarkers, we compared the differentially expressed miRNAs within the perilymph and compared it to serum miRNA profiles in patients with active bilateral Menière's disease and healthy age matched controls.

## Methods

Human perilymph sampling was approved by the University of Kansas Human Studies Committee and Institutional Review Board (Study00142630). All experiments performed were in accordance with relevant guidelines and regulations. Patients were recruited for perilymph sampling if they were undergoing a surgical procedure where the inner ear was already being opened. miRNA perilymph profiling was performed using two disease classes, Menière's disease and otosclerosis. For Menière's disease, perilymph was collected at the time of labyrinthectomy for patients with intractable symptoms and who failed hearing conservative management. Perilymph sampling in patients with a healthy and normal ear is not currently feasible, therefore we used otosclerosis patients as a control since they have minimal SNHL. Patients with SNHL secondary to otosclerosis were not included. All patients received standard of care and underwent surgical treatment with either stapedotomy or labyrinthectomy. When the patient's inner ear was opened for their indicated procedure, a small sterile glass capillary tube was used to collect ~2–5 μL volume of perilymph (11).

In the second part of this project, we sought to evaluate if the perilymph profile of Meniere's disease is also reflected in the serum of patients. An overlap would indicate that miRNAs may serve as a promising biomarker for Menière's disease. In order to evaluate serum miRNA profiles, human serum was collected, and miRNAs were isolated from five patients with active bilateral Menière's disease. Serum miRNA profiles from Menière's disease were compared to five healthy age matched controls.

### Patient Selection and Audiometry

All patients underwent pure tone audiometry prior to their respective procedures. Air and bone conduction thresholds were determined, and a CT scan of the temporal bone was performed for all patients with diagnosis of otosclerosis. Only patients with conductive hearing loss (CHL) and radiologically confirmed otosclerosis were included in the CHL control group (*n* = 5). For the Meniere's disease cohort, patient's required the diagnosis of “definite Meniere's” based on the Bárány Society and adopted by the American Academy of Otolaryngology Head and Neck Surgery (13, 14). This included documented low to mid frequency SNHL, episodic vertigo that lasts 20 min to 12 h, and aural symptoms (fullness and/or tinnitus). Patients were diagnosed with bilateral Meniere's disease if they fit “definite Meniere's,” where symptoms lateralized to both ears (tinnitus, aural fullness, pressure) and documented low frequency SNHL in both ears at some point in their Meniere's history. A total of five patients (*n* = 5) underwent labyrinthectomy for poorly controlled Menière's disease who had failed hearing conservative therapy including dietary control, diuretics, betahistine, and/or endolymphatic sac decompression. Patients were not considered for intratympanic gentamycin injections.

### Perilymph Sampling During Stapedectomy

The skin of the external auditory canal was injected with 0.5 ml of 1% lidocaine + 1:100,000 epinephrine. Using a round knife, a cut was made in the skin of the external canal and small tympanomeatal flap was carefully elevated medially revealing the middle ear structures. Using the Omniguide™ CO_2_ laser with a power setting of four watts, 0.1 s single bursts, the stapes superstructures were removed. Using the laser, a rosette fenestration was made in the stapes footplate. Upon making the fenestration, perilymph could be seen coming out from the vestibule. We then removed excess perilymph with a sterile glass capillary tube. After successful collection of perilymph fluid, the stapes footplate fenestration was enlarged to accommodate the stapes prosthesis. The surgery was then completed in a standard fashion.

### Perilymph Sampling During Labyrinthectomy

Through a post auricular incision, a standard mastoidectomy was performed and the horizontal semicircular canal was identified. The horizontal semicircular canal was carefully blue lined with a diamond drill and opened on one posterior end to allow free flow of perilymph out of the inner ear. The perilymph sample was obtained using a sterile glass capillary tube. A standard labyrinthectomy was performed following collection of adequate sample.

### Human Serum Sampling

Five patients with bilateral active Menière's disease based on 2015 Barany Society diagnostic criteria and five age matched healthy controls were enrolled. Six ml of whole blood was collected from each participant and transferred to tubes containing EDTA. The samples were then spun down at 2,000 × g for 15 min. The serum layer was then pipetted off and frozen at −20°C. Once all 10 samples were collected, microRNA was isolated from the serum using Qiagen miRNeasy Serum/Plasma Kit (Cat No./ID 217184).

### MicroRNA Analysis

Perilymph collection has been previously described (11, 23). Total RNA was extracted with Trizol reagent (Thermofisher, cat #15596018) and purified by centrifuging with a phase lock heavy gel (Tiagen, cat # WMS-2302830). MicroRNA from serum samples was extracted as described above. Samples from both perilymph and serum were analyzed with an Agilent RNA6000 Pico kit using an Agilent Bioanalyzer 2100 yielding on average 0.5–2 ng of total RNA per sample. Samples were processed and analyzed with an Affymetrix miRNA 4.0 array to determine the presence of micro RNAs as per manufacturer instructions. The Affymetrix miRNA 4.0 array interrogates all miRNA sequences listed in miRBase Release 20; interrogating 30,434 mature miRNAs from 203 organisms of which 2,578 are from humans. The arrays were background corrected, normalized, and gene-level summarized using the Robust Multichip Average (RMA) algorithm. In order to ascertain which miRNAs were significantly expressed in each array, for each miRNA probe in the array, a detection *p*-value was computed based on a Wilcoxon Rank-Sum test of the miRNA probe set signals compared to the distribution of a GC matched background signal comprising of anti-genomic probes in the same array. The detection *p*-values were adjusted for multiple hypothesis testing (FDR) using the Benjamini and Hochberg method. These analyses were performed using Affymetrix Expression Console Software. miRNAs with a normalized log2 signal intensity ≥ 7 and an adjusted detection *p*-value (FDR) ≤ 0.05 were considered significantly expressed in the assay.

To further understand miRNA interaction within the human cochlea as it relates directly to Menière's disease, we approached analysis in a two-step process. First, we compared the perilymph miRNA profiles between patients with Menière's disease and otosclerosis (control) to differentiate which miRNAs are unique to Menière's disease using Qlucore software (Qlucore Inc., Lund Sweden). The miRNA expression data were then analyzed using Ingenuity Pathway Analysis (IPA) software (Qiagen Bioinformatics, Redwood City, CA). In order to further understand miRNA interactions within the human inner ear, we used IPA software to predict potential interactions with genes expressed in a normal hearing human cochlea (GEO Series accession number GSE128505) (10, 29). Secondly, in order to identify miRNAs that are clinically relevant, we screened the identified miRNA-mRNA interactions for genes that have been identified to play a pathologic role in Meniere's disease in either animal models or population studies (30–33). Key miRNAs identified were then compared the miRNA expression levels in the serum of patients with Meniere's disease and controls. MicroRNA data from serum samples was analyzed by microarray as described above with a focus on miRNAs that had been identified as differentially expressed in serum.

### Serum miRNA qPCR Validation Analysis

After running microRNA arrays from the serum samples, residual RNA from the samples was validated by Q-RT-PCR. Two serum samples were excluded from further processing due to low RNA concentration, one control and one from the Meniere's cohort. A second sample from the Meniere's cohort was excluded from analysis due to genomic DNA contamination, leaving 4 samples in the control group and 3 in the Meniere's group. cDNA was synthesized using the miScript II reverse transcription kit (Qiagen, cat#218161). qPCR was then performed on the BioRad CFX using the miScript SYBR Green miRNA PCR system (Qiagen, cat#218073). All reactions were performed in triplicate and the Ct value was determined using the threshold calculated by the BioRad software. Mir-191-5p was used for normalization based on its stable expression across treatment and control samples. The universal primer from the miScript SYBR Green miRNA PCR kit (Qiagen, cat#218073) was used as the reverse primer for each reaction. MiScript primer assays for hsa-miR-191-5p (5′ CAACGGAATCCCAAAAGCAGCTG 3′) and hsa-miR-1270 (5′ CTGGAGATATGGAAGAGCTGTG 3′) were used as forward primers for qPCR (Qiagen, cat#218300). The hsa-miR-1299 forward primer sequence was 5′ CTGGAATTCTGTGTGAGGG 3′. Fold change was calculated using the 2^−ΔΔCT^ method (32).

## Results

### Perilymph miRNA Profile in Meniere's Disease vs. Otosclerosis (CHL/Controls)

In order to identify which miRNAs are unique to Meniere's disease, we compared the perilymph miRNA expression profiles between Meniere's disease (*n* = 5) and otosclerosis (*n* = 5) (Table 1). One significant challenge is that perilymph sampling in patients with normal hearing is not currently feasible. Therefore, patients with otosclerosis serve as our control, given this pathology is largely exclusive to the stapes footplate. To maximize this patient population as controls, we excluded patients with SNHL secondary to advanced otosclerosis. Using gene expression analysis, we identified 16 unique miRNAs that were significantly downregulated in patients with Menière's disease (Table 2).

**Table 1:** Audiometric data for both patient cohorts.

	**Meniere's disease**	**Otosclerosis**
Number of patients	5	5
Age (years) (Mean ± Std)	66.6 ± 18.1	52.2 ± 10.6
**PTA (dB) (Mean** **±** **Std)**		
Air	69.2 ± 18.8	58 ± 6.7
Bone	60.8 ± 23.2	30.4 ± 8.6

**Table 2:** MiRNAs significantly downregulated in Menière's disease vs. otosclerosis perilymph.

**Micro RNA**	***T*-Test**
hsa-miR-4296	−2.146997624
hsa-miR-6827-3p	−2.156687123
hsa-miR-3658	−2.205593839
hsa-miR-219b-5p	−2.22463251
hsa-miR-1243	−2.231753534
hsa-miR-4715-3p	−2.258995064
hsa-miR-5700	−2.277212392
hsa-miR-4746-5p	−2.298984529
hsa-miR-542-3p	−2.307117824
hsa-miR-1299	−2.332907361
hsa-miR-1255a	−2.438288476
hsa-miR-6853-5p	−2.443161671
hsa-miR-518f-3p	−2.521748132
hsa-miR-620	−2.556715548
hsa-miR-5192	−2.680462648
hsa-miR-424-3p	−3.009047712

The 16 miRNAs altered in Menière's disease were screened for targets in a human cochlear cDNA library using IPA software. Analysis was only limited to miRNA-mRNA prediction interactions that have either been experimentally proven or “high probability” based on paired nucleotide sequencing between miRNA and mRNA. The 16 miRNAs unique to Meniere's disease were predicted to interact with over 220 genes within the cochlea. To focus our search, we only analyzed proposed gene candidates that have been proven to play a pathologic role in Meniere's disease through either animal models or human genetic population studies. Aberrant aquaporin regulation have been consistently linked to Menière's disease in both experimental models and population studies (33, 34). Altered regulation of both pathways have been linked to Menière's disease as well (18, 35–37). Six of the 16 miRNAs are predicted to regulate aquaporin gene expression (Table 3). Twelve of the 16 miRNAs are predicted to regulate either inflammatory or autoimmune regulatory pathways (Table 4).

**Table 3:** MiRNAs unique to Meniere's disease perilymph that are postulated to directly regulate aquaporin gene expression.

**Micro RNA**	**Aquaporin**
hsa-miR-219b-5p	AQP2
hsa-miR-1243	AQP4
hsa-miR-4746-5p	AQP2 + AQP4
hsa-miR-1299	AQP2
hsa-miR-1255a	AQP3 + AQP4
hsa-miR-1270	AQP4

**Table 4:** MiRNAs differentially expressed in Meniere's disease perilymph and their inflammatory pathway targets.

**Micro RNA**	**Inflammatory pathway targets**
hsa-miR-542-3p	KRTAP6-3, LINC01169, R3HDM2
hsa-miR-6853-5p	POLR2J2/ POLR2J3, NME4
hsa-miR-424-3p	OTOR, HIGD1A, RNF13
hsa-miR-1243	RNF13, LYRM2, RPS16, RAP1B
hsa-miR-5700	TNFRSF11B, RAP1B, ELOC
hsa-miR-518f-3p	RAP1B
hsa-miR-4746-5p	NMNAT2, STEAP2
hsa-miR-1255a	RPS16, NMNAT2
hsa-miR-4715-3p	SCP2
hsa-miR-1299	SCP2, ATP5PO
hsa-miR-3658	ATP5PO, SCP2
hsa-miR-1270	SCP2, STEAP2

### Evaluating Human Serum miRNA Profiles for Potential Biomarkers

An ideal candidate Menière's disease biomarker would be identifiable within both the perilymph and serum of patients, thereby allowing us to evaluate the function of the inner ear based on a serum sample. We collected and isolated miRNAs in the serum of patients with bilateral active Menière's disease (*n* = 5) and age matched controls (*n* = 5). Using miRNA array analysis, we identified 79 alternately expressed miRNAs in the serum of patients with Menière's disease compared to controls. miRNA-1299 and−1270 were uniquely and differentially expressed in the perilymph and serum of patients with Menière's disease, making both promising biomarkers. Both miRNA-1299 and-1270 were linked to inflammatory and autoimmune pathways. miRNA-1299 can be linked to aquaporin expression.

To further evaluate if either miRNA-1299 or−1270 could be utilized as a Menière's disease biomarker, qPCR was utilized to validate if both were unique and exclusive to Meniere's disease serum. Using the 2^−ΔΔCT^ method, miRNA-1299 downregulated 3.13-fold in the serum of patients with Menière's disease compared to controls. However, miRNA-1270 had similar expression levels in the serum of both patient cohorts (fold change = 0.923).

## Discussion

There is a need for a surrogate marker for Meniere's disease, to not only serve as a diagnostic marker but also help further elucidate what may be occurring on a molecular level. This study is one of the first studies to describe the miRNA profiles in patients with Menière's disease. By evaluating and comparing miRNA perilymph and serum profiles in patients with Menière's disease to controls, miRNA-1299 was identified exclusively in patients with Menière's and may serve as a promising disease specific biomarker.

miRNAs have shown significant potential as a reliable biomarker for otherwise difficult to diagnose diseases (26, 38, 39). miRNAs are small non-coding RNAs that downregulate gene expression through various negative feedback loops (39). Using IPA software, we are able to identify potential miRNA—mRNA interactions in a human cochlear cDNA library to gain further insight into various aberrant pathways in Menière's disease. However, this type of analysis is limited. We can only interrogate and infer mRNA regulation, and we do not directly identify genes involved with Menière's disease (24). Despite this limitation, we know miRNAs play a key mechanistic role in various inner ear pathologies such as sudden hearing loss and inner ear development (26, 27, 29, 30). Additionally, miRNAs have been identified within all main cellular compartments, exosomes, and cell free components throughout the body and have been successfully used as biomarkers for other neurodegenerative diseases like Alzheimer's and Parkinson's (25, 36, 37). Finally, in previous work, we found miRNAs exhibit inner ear disease specific profiles and that various inner ear pathologies can be diagnosed based on miRNA profiles alone (11, 22, 23). Taken together, miRNAs offer an intriguing biomarker for Menière's disease.

We identified 16 unique miRNAs that were significantly downregulated in the perilymph of patients with Menière's disease. While these 16 miRNAs were proposed to regulate over 220 different genes within the cochlea, we narrowed our focus to gene pathways that have been shown to play a pathologic role in Menière's disease. Aquaporins are a group of intramembrane proteins that serve as water channels in various cellular processes and have been shown to potentially play a key pathogenic role in Menière's disease (21, 40). In guinea pig models, endolymphatic hydrops can be induced through administration of vasopressin, resulting in upregulation of aquaporins (33). In humans, AQP2 and AQP3 have been shown to be aberrantly expressed in genetic population studies (34). It was promising that not only unique miRNAs were identified in Menière's patient cohorts, but that their genetic interactions are linked to pathways that have been identified in Menière's disease. IPA software predicted that miRNA-1255 and−1243 target to AQP3 mRNA, and miRNA-4746 and−1299 are target AQP2 mRNA. Compared to previous miRNA perilymph studies on sudden and progressive sensorineural hearing loss, there was no overlap observed in the 16 significant miRNAs identified in this study (29, 30).

Twelve of the 16 miRNAs unique to Menière's disease are directly predicted to regulate inflammation and/or autoimmune pathways. Recent research supports the link between inflammation, cellular degeneration, and the pathogenesis of Menière's disease (16). By analyzing the perilymph proteome in patients with Menière's disease, Lin et al. discovered 38 proteins that were differentially expressed in patients with Menière's disease and strongly linked to tissue injury and inflammation (10). Several pathologic mechanisms have been proposed for Menière's diseases involving inflammatory pathways including viral-induced inflammation (10), otitis media induced inflammatory products and toxins (41), circulating immune complexes leading to increase cellular permeability (37, 42), genetic predisposition to altered NF-KB mediated inflammatory response (43), distinct and altered cytokine profiles (44), and autoimmunity (37, 45). Together, these studies suggest a strong correlation between Meniere's disease and an aberrant inflammation cascade. Genes that were predicted to be targeted by two or more of the twelve miRNAs associated with inflammation included *TNFRSF11B, RAP1B, NMNAT2, RPS16, R3HDM2, STEAP2, and SCP2*. Altered TNF expression has been proposed to play a significant role in Meniere's disease and TNF-α inhibitors are being preliminarily investigated as a therapeutic intervention (45, 46). From our identified miRNAs, miRNA-1299 was identified as the most promising biomarker for Menière's disease since it is differentially regulated in both perilymph and serum of Menière's patients, and is predicted to target *R3HDM2, SCP2*, and *ATP5PO* mRNA.

While perilymph and serum miRNAs have been uniquely linked to Menière's disease and miRNA-1299 shows potential as viable disease biomarker, there are several limitations to this study. First, by analyzing miRNAs within the perilymph, we are studying a small subset of miRNAs that are limited to the extracellular exosomes or cell free miRNA and not evaluating tissue directly (47). Secondly, although promising miRNAs were identified, the small sample size severely limits statistical analysis and ability to draw stronger conclusions. Future prospective studies will need to increase the sample size and evaluate these miRNA biomarkers as compared to other inner ear pathologies that can present simultaneously with Menière's disease, such as sensorineural hearing loss. For example, future studies will need to compare Meniere's miRNA profiles to patients with known isolated genetic causes of SNHL, such as patients with the DFNA9 mutation. Third, we anticipate that larger cohorts may also reveal that different subtypes of Meniere's disease as suggested by epidemiological studies (48). Furthermore, if these miRNA biomarkers remain promising in larger studies, future research endeavors will need to validate their mechanism of action. As further samples are collected for disease specific biomarkers, it will be critical to not only collect perilymph and serum samples from the same patients, but also compare miRNA disease profiles in patients with unilateral and bilateral active Menière's disease. Finally, statistically significant miRNAs identified in this study are different from our machine learning studies (31). We hypothesize differences are likely attributed to machine learning methodology, which is focused on miRNA complex pattern matching and not solely differences in single miRNA expression levels. Approaches in this study helped find statistically significant miRNAs based on expression alone, whereas machine learning may find a subset of miRNAs and how their expression pattern, both upregulation and downregulation, may be interconnected and unique to various inner ear pathologies. Future work will need to further understand the validity of both methodological approaches and how they can help identify single miRNAs as compared to assay like expression patterns of miRNA biomarkers for inner ear pathologies.

## Conclusion

The pathophysiology and disease mechanisms driving Menière's disease remain unclear. While a direct tissue biopsy of the inner ear is not feasible, perilymph sampling has shown significant promise as a “liquid biopsy.” In this study, Menière's disease not only demonstrated a unique and distinct miRNA profile compared to controls, but unique miRNAs can be directly linked to proven or well-accepted aberrant disease mechanisms underlying Menière's disease. miRNA-1299 is differentially expressed in the perilymph and serum of patients with Menière's disease, predicted to regulate both aquaporin and inflammatory disease mechanisms linked to Menière's disease, and may ultimately serve as a promising biomarker. While definitive conclusions are limited by a small sample size, miRNA profiling in patients with Menière's disease is a feasible methodology to further elucidate what may be occurring a molecular level and identify future biomarkers to diagnose, prognose, and guide treatment.

## Data Availability Statement

The datasets presented in this study can be found in online repositories. The names of the repository/repositories and accession number(s) can be found at: https://www.ncbi.nlm.nih.gov/geo/query/acc.cgi?acc=GSE176560.

## Ethics Statement

The studies involving human participants were reviewed and approved by University of Kansas Medical Center Institutional Review Board. The patients/participants provided their written informed consent to participate in this study.

## Author Contributions

MS, HW, MSP, and HS planned and performed the experiments. MS, HW, and HS gathered patient perilymph and serum samples. MS, HS, HW, AW, and MSP contributed to the interpretation of data and drafting of the manuscript. All authors contributed to the article and approved the submitted version.

## Conflict of Interest

The authors declare that the research was conducted in the absence of any commercial or financial relationships that could be construed as a potential conflict of interest.
